# Novel Insights into Ethanol-Soluble Oyster Peptide–Zinc-Chelating Agents: Structural Characterization, Chelation Mechanism, and Potential Protection on MEHP-Induced Leydig Cells

**DOI:** 10.3390/md22100465

**Published:** 2024-10-10

**Authors:** Zhen Lu, Qianqian Huang, Xiaoming Qin, Fujia Chen, Enzhong Li, Haisheng Lin

**Affiliations:** 1 Guangdong Provincial Key Laboratory of Aquatic Products Processing and Safety, College of Food Science and Technology, National Research and Development Branch Center for Shellfish Processing, Guangdong Provincial Engineering Technology Research Center of Seafood, Guangdong Ocean University, Zhanjiang 524088, China; 1122103003@stu.gdou.edu.cn (Z.L.); huangqq339@163.com (Q.H.); 2 School of Biological and Food Processing Engineering, Huanghuai University, Zhumadian 463000, China; shenfujiawlqs@163.com (F.C.); enzhongli@163.com (E.L.); 3 Collaborative Innovation Center of Seafood Deep Processing, Dalian Polytechnic University, Dalian 116034, China

**Keywords:** zinc-chelating peptides, MEHP, TM3, in silico screening, apoptosis

## Abstract

Numerous studies have reported that mono-(2-ethylhexyl) phthalate (MEHP) (bioactive metabolite of Di(2-ethylhexyl) phthalate) has inhibitory effects on Leydig cells. This study aims to prepare an oyster peptide–zinc complex (PEP-Zn) to alleviate MEHP-induced damage in Leydig cells. Zinc-binding peptides were obtained through the following processes: zinc-immobilized affinity chromatography (IMAC-Zn^2+^), liquid chromatography–mass spectrometry technology (LC-MS/MS) analysis, molecular docking, molecular dynamic simulation, and structural characterization. Then, the Zn-binding peptide (PEP) named Glu—His—Ala—Pro—Asn—His—Asp—Asn—Pro—Gly—Asp—Leu (EHAPNHDNPGDL) was identified. EHAPNHDNPGDL showed the highest zinc-chelating ability of 49.74 ± 1.44%, which was higher than that of the ethanol-soluble oyster peptides (27.50 ± 0.41%). In the EHAPNHDNPGDL-Zn complex, Asn-5, Asp-7, Asn-8, His-2, and Asp-11 played an important role in binding to the zinc ion. Additionally, EHAPNHDNPGDL-Zn was found to increase the cell viability, significantly increase the relative activity of antioxidant enzymes and testosterone content, and decrease malondialdehyde (MDA) content in MEHP-induced TM3 cells. The results also indicated that EHAPNHDNPGDL-Zn could alleviate MEHP-induced apoptosis by reducing the protein level of p53, p21, and Bax, and increasing the protein level of Bcl-2. These results indicate that the zinc-chelating peptides derived from oyster peptides could be used as a potential dietary zinc supplement.

## 1. Introduction

At present, reproductive damage caused by environmental factors has attracted the attention and research of a large number of scholars. Among them, the types of endocrine disruptors are increasing, and several studies have shown that male infertility is closely related to exposure to environmental endocrine disruptors (EDCs) [1]. Di(2-ethylhexyl) phthalate (DEHP) is a common EDC that is widely used in plastic products worldwide. A large number of studies have reported that DEHP and MEHP (bioactive metabolite of DEHP) harm multiple organs and induce endocrine disruption and testicular damage [2].

DEHP enters into the body and is metabolized into MEHP by mammalian intestinal hydrolytic enzymes. It is more toxic than DEHP and has reproductive and developmental toxicity [3,4,5]. The chemical structures of DEHP and MEHP are similar to those of hormones in the body and can competitively bind the hormone receptor, finally disrupting the maintenance of normal levels of hormones related to the body [6]. Research has found that workers who were exposed to DEHP in their daily lives had higher levels of the urinary metabolite MEHP, which was significantly negatively correlated with serum testosterone levels [7]. Recently, numerous studies have shown that MEHP exposure can induce oxidative stress and play a crucial role in the occurrence and development of cell apoptosis [2,8,9]. Previous studies have found that TM3 cells activated the p53 pathway after treatment with DEHP/MEHP, and the inhibition of p53 and p21 by pifithrin-α significantly reduced MEHP-induced injuries in TM3 cells [10]. DEHP has been found to cause morphological changes in the testis, including oxidative stress, apoptosis, necrosis, and the loss of spermatogenic cells, which is related to the depletion of zinc in the testis [11]. Phthalates impaired the reproductive function in male rats, while zinc supplementation inhibited the reduction of serum T, LH, and FSH levels [12].

Zinc can protect organisms from free radical attacks by maintaining levels of metallothionein, and it can contribute to the synthesis of SOD [13]. But phytase and dietary fiber are prone to combine with divalent cations such as zinc to form insoluble precipitates, which affects their bioavailability in vivo [14]. Research has found that the long-term use of zinc salts can stimulate digestion and absorption in the gastrointestinal tract [15]. Metal element-bound peptides are new compounds produced through reactions between peptide chains and metal ions. Accumulating evidence demonstrates that the bioavailability of peptide–zinc chelates was superior to zinc salts [16]. Research has found that in an environment that simulates gastrointestinal digestion in vitro, metal-binding peptides with high zinc-chelating ability still maintain approximately 85% of their original biological activity [17]. It has also been found that oyster peptide-chelated zinc can be used as a novel zinc supplement to ameliorate prostatitis in rats, resulting in significant increases in superoxide dismutase, glutathione activity, and serum zinc concentrations [18]. The protective effects of the selenium-containing peptide RYNA (Se) MNDYT (Se-P2) and its parent peptide (RYNAMNDYT (P2)) derived from selenium-rich Cordyceps militaris were compared in mice injured by LPS, and the Se-P2 treatment showed better antioxidant effects than the P2 treatment [19]. Chen et al. isolated a calcium-chelating peptide of DGDDGEAGKIG from the tilapia scale protein, which significantly improved the physical and biomechanical properties of the femur in calcium-deficient rats [20]. But simultaneous analysis of the potential protection of the peptide–zinc complex on MEHP-induced Leydig cells and the Zn-binding capacity of ethanol-soluble oyster peptides (OP-ESs) is limited.

Regarding the processing of peptides to enhance the physiological activity of metal peptides, it was reported that the tuna flesh hydrolysate from an ethanol-soluble fraction had higher xanthine oxidase inhibitory activity [21]. Similarly, Yuan et al. found that the isolated ethanol-soluble cod peptides can regulate the immune activity of both innate and adaptive lineages [22]. Kong et al. also found that ethanol-soluble soybean protein hydrolysates had better metal-binding capacity [23]. In this research, 85% ethanol was used to make ethanol-soluble oyster peptides (OP-ESs), and then the zinc-binding peptides were isolated by IMAC and identified by liquid chromatography–tandem mass spectrometry (LC-MS/MS). The high zinc-binding peptides were selected using molecular docking, and the conformational stability of the peptide–zinc complexes was certified by MD simulations. Finally, the screened peptides chelated with zinc were determined using in vitro cell experiments for the potential protection on MEHP-induced Leydig cells, and to expand its application successfully as a zinc supplement in the future.

## 2. Results

### 2.1. Isolation of Zinc-Binding Peptides Using IMAC and Determination of Amino Acidic Profiles and Molecular Weight Distribution

**Figure 1:**
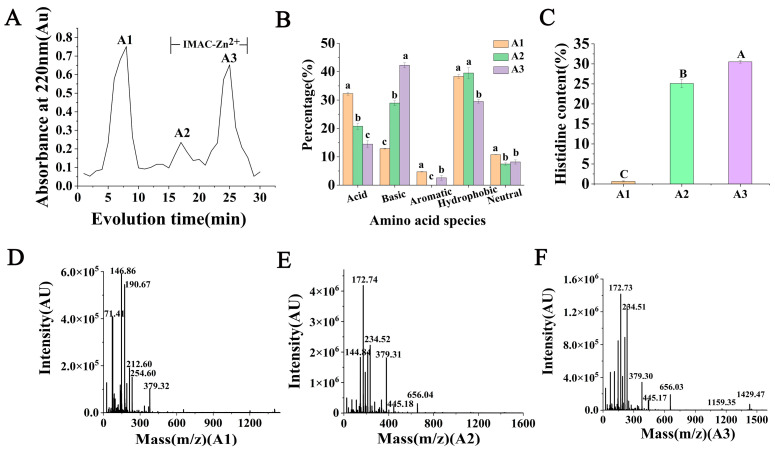
Isolation of zinc-binding peptides using IMAC and determination of amino acidic profiles and molecular weight distribution. (**A**) A1, A2, A3 were eluted by IMAC-Zn^2+^ from OP-ES; (**B**) ratio of different amino acids of A1, A2, A3; (**C**) percentage of histidine amino acid of A1, A2, A3. MALDI-TOF-MS spectrum of peptides obtained from isolated fractions of IMAC ((**D**): A1, (**E**): A2, (**F**): A3). Different lowercase letters (a–c) on top of the bars in same amino acid specie denote significant difference (*p* < 0.05). Different capital letters (A–C) indicated the between-group significant differences (*p* < 0.05).

IMAC-Zn^2+^ is a highly selective purification technique used to separate zinc-chelating peptides with high concentrations of histidine [24]. In Figure 1A, A1 was eluted first with equilibrating buffer (20 mM PBS, pH = 7.4) with no zinc-binding capacity. A2, which was first eluted with the PBS buffer (20 mM, pH = 4.0) quickly, was due to the weakly bound peptides. A3, the fraction of interest and with strongly bound zinc-binding peptides, was eluted last.

In the present study, zinc-binding peptides were isolated from the OP-ES, and the fractions containing different amino acids were evaluated. Amino acid analysis of A1, A2, and A3 is shown in Figure 1B,C. The residues of amino acids are divided into the following five categories: acidic amino acid, basic amino acid, aromatic amino acid, hydrophobic amino acid, and neutral amino acid. The results showed that the residues of basic amino acid were critical in the peptide–zinc-binding process, with a high percentage in A3. The content of histidine from A1, A2, and A3 was 0.66%, 25.10%, and 30.50%.

As shown in Figure 1D, the molecular weight distribution of A1 ranged from 71.41 to 379.32 Da, and it was mainly dominated by free amino acids and small molecular weight peptides. The molecular weight distribution of A2 ranged from 144.84 to 656.04 Da, as shown in Figure 1E. The molecular weight distribution of A3 ranged from 172.73 to 1429.47 Da, as shown in Figure 1F. As can be seen from the above experimental results, the number of polypeptide fragments with molecular weights between 1000 and 1500 Da in A3 increased with the enhancement of the binding ability to zinc ions.

### 2.2. The Protective Effect of Isolated and Purified Peptides and Their Zinc Chelates in MEHP-TM3 Cells

**Figure 2:**
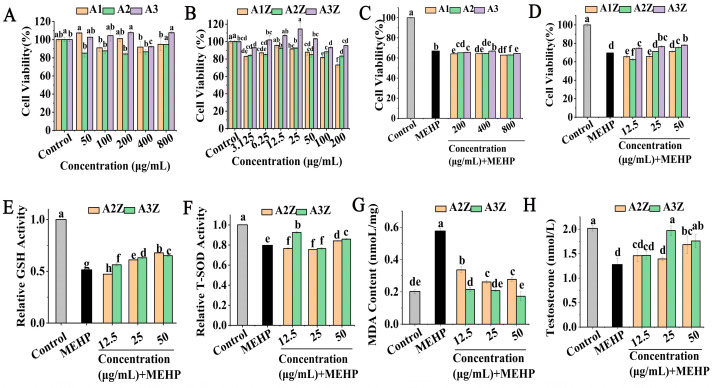
The protective effect of isolated and purified peptides and their zinc chelates in MEHP-TM3 cells. Effect of different IMAC fractions (**A**) and their zinc chelates (**B**) on the viability of TM3 cells; effect of different IMAC fractions (**C**) and their zinc chelates (**D**) on the viability of MEHP-injured TM3 cells. Effects of peptide–zinc chelates (A2Z, A3Z) on MEHP-induced oxidative stress and reproductive hormones in TM3 cells. (**E**) GSH level; (**F**) T-SOD level; (**G**) MDA level; (**H**) T level. Different letters represented the significant difference at *p* < 0.05. Values are mean ± standard error of at least 3 independent experiments.

As shown in Figure 2A, the peptide of A1, A2, and A3 showed no cytotoxicity to TM3 cells within 50–800 μg/mL. In general, more than 80% of cell viability has been considered non-cytotoxic with peptide treatment on TM3 cells [25]. A1Z, A2Z, and A3Z showed no significant cytotoxicity within the concentrations of 3.125–100 μg/mL, and the effect of A3Z was better than that of A1Z, A2Z (Figure 2B). As shown in Figure 2C, the result was that A1, A2, and A3 had no protective effect on TM3 cells damaged by MEHP. However, the cell viability of the MEHP-injured group decreased to 69.60 ± 0.14% (*p* < 0.05), the cell viability of the A1Z, A2Z, and A3Z pretreatment groups was 71.15 ± 0.92%, 75.30 ± 1.38%, and 78.06 ± 0.67%, respectively (Figure 2D), and A3Z had the best protective on MEHP-injured TM3 cells.

Researchers also found that low-dose MEHP disrupted germ cells, supporting cell differentiation and mesenchymal cell function in rat fetal testis with oxidative damage to the testis [26]. The results of A2Z and A3Z on the GSH enzyme activity of the damaged TM3 cells are shown in Figure 2E. Compared with the MEHP group, the GSH activities in TM3 cells treated with A2Z decreased by 8.26% in the 12.5 μg/mL condition, and increased by 18.54% and 31.76% in the 25 μg/mL and 50 μg/mL conditions. Meanwhile, the GSH activities in TM3 cells increased by 9.40%, 22.47%, and 26.55%, respectively, after treatment with A3Z (12.5, 25, and 50 μg/mL). Moreover, the effects of the zinc–peptide complex on the T-SOD activity in TM3 cells are shown Figure 2F; the T-SOD activity of the cells treated with A2Z decreased by 3.90%, and the group treated with A3Z increased by 16.05% at a concentration of 12.5 μg/mL, compared with the model group. As shown in Figure 2G, the MDA levels of the model group were 0.577 ± 0.016 nmol/mg of protein (*p* < 0.05), and the MDA levels in TM3 cells pretreated with A3Z (0.215 nmol/mg of protein) were significantly lower than those in the A2Z group (0.337nmol/mg of protein) at 50 μg/mL. From the results above, it can be seen that A2Z and A3Z could inhibit the MEHP-induced oxidative stress damage in TM3 cells by increasing the enzymatic antioxidant system activity and decreasing the MDA content, among which A3Z with higher zinc content had the best effect.

The current results show that the testosterone levels in the MEHP group were significantly lower than those of the controls in Figure 2H. Compared with the model group, A2Z increased by 11.36% and 34.66% under the middle and high concentration, and A3Z increased by 57.67% and 40.71%, respectively. Among the results above, when administered at the same concentration or at a low concentration, A3Z showed stronger antioxidant activity and better effects.

### 2.3. Identification of A3 In Silico Prediction and Molecular Docking

Computerized analysis and in silico analysis have been widely applied to develop a wide range of bioactive metal-chelating peptides. Target peptides were screened for further identification via in silico analysis. Table 1 presents the list of candidates identified from A3, showing some properties of the screening peptide, such as the instability index, toxicity, and estimated solubility. These 12 peptides were expected to have good solubility and none of them were toxic. If the instability index is less than 40, it is predicted to be stable [27], and the 12 obtained peptides were all stable. Meanwhile, the molecular weight of A3 ranged from 959 Da to 1500 Da.

As shown in Table 2, the selected 12 peptides can bind to zinc ions, which have at least one binding site, some with three or four. Asparticacid (Asp), histidine (His), and Asparagine (Asn) occur most frequently at these chelating sites. PEP3, PEP6, PEP9, PEP10, and PEP11 have high docking energy, which demonstrated good chelation of peptides with Zn^2+^. So, these five peptides were selected for further research.

### 2.4. Determination of Zinc-Chelating Ability and Molecular Dynamics Simulation

**Figure 3:**
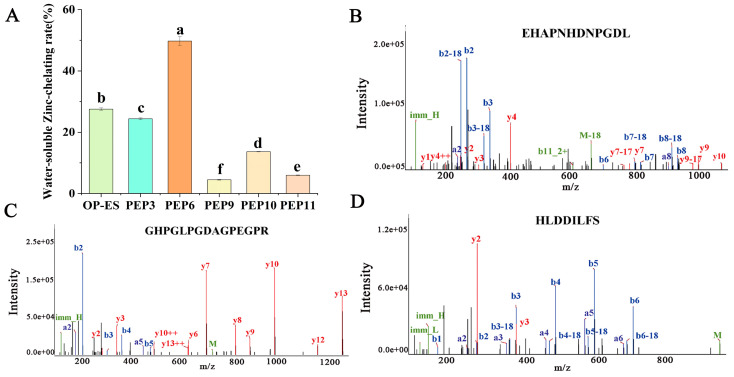
Screening and identification of zinc-chelating peptides. (**A**) Zinc-chelating ability of synthetic peptides; mass spectra of the peptides EHAPNHDNPGDL (**B**), GHPGLPGDAGPEGPR (**C**), and HLDDILFS (**D**) identified in A3. Different letters represented the significant difference at *p* < 0.05.

To evaluate the potential of the five peptides as metal-chelating agents, their chelating ability for Zn^2+^ was determined. In Figure 3A, from high to low zinc-chelating ability, is EHAPNHDNPGDL (49.74 ± 1.44%) > GHPGLPGDAGPEGPR (24.41 ± 0.29%) > HLDDILFS (13.67 ± 0.08%) > YHDHDVPCA (5.99 ± 0.08%) > DVHPEHPY (4.50 ± 0.10%). EHAPNHDNPGDL (PEP6) exhibited the highest zinc-binding activity and was higher than that of the OP-ES (27.50 ± 0.41%). In addition, peptides GHPGLPGDAGPEGPR (PEP3) and HLDDILFS (PEP10) also had higher zinc-binding activity among the five synthetic peptides, and the mass spectra of these three peptides are shown in Figure 3B–D.

**Figure 4:**
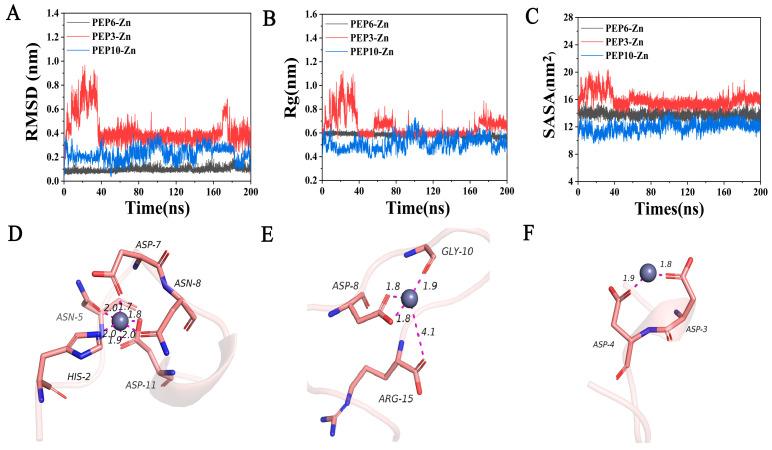
Graphical representation of the molecular dynamics simulation studies conducted during 200 ns and binding mode of zinc-chelating peptides to Zn^2+^ after molecular dynamics simulation. (**A**) Root mean square deviation (RMSD); (**B**) radius of gyration (Rg) curves; (**C**) solvent-accessible surface area (SASA). (**D**) PEP6-Zn; (**E**) PEP3-Zn; (**F**) PEP10-Zn. The atoms represented by different colors are as follows: red: O; blue: N; pink: C; gray: Zn^2+^.

To further evaluate the molecular docking results, molecular dynamics simulations were performed within 200 ns. As shown in Figure 4A, the RMSD of PEP6-Zn stabilized at about 1 Å. The RMSD value of PEP6-Zn was lower than that of PEP10-Zn and PEP3-Zn. Rg is usually used to describe the compactness of protein structures [28]. During the entire 200ns simulation process, the Rg value of the PEP6-Zn complexes was limited to approximately 6 Å (Figure 4B). According to Figure 4C, it can be seen that the SASA of the PEP3-Zn complex fluctuated a lot, but after a long time of movement, the stability was improved, and the SASA of the PEP10-Zn complex decreased significantly and the stability was better, while the SASA of PEP6-Zn remained at 14 nm^2^, which was directly related to the structure of the peptide [29].

This figure reflected the precise binding mode of peptides and zinc after molecular dynamics simulation. According to Figure 4D–F, the zinc ion is docked with peptides in three forms. The first form is the binding of zinc with negatively charged carboxyl groups (Asp-11 and Asp-8). The second form is the binding of zinc with C=O in the carboxyl groups (Asn-5, Asp-7, Asn-8, Asp-11, Asp-8, Arg-15, Gly-10, Asp-3, and Asp-4). The third form is the binding of zinc to the nitrogen atom on the imidazole group (His-2). This result indicated that the carboxyl group and the amino group bound to the zinc. The docking conformation of PEP6-Zn suggested that the dodecapeptide formed five binding bonds (Asn-5, Asp-7, Asn-8, His-2, and Asp-11) with zinc. This indicated that PEP6-Zn has a tight affinity for zinc. PEP3-Zn formed four binding bonds with zinc and PEP10-Zn formed two binding bonds with zinc.

The binding free energy of the PEP6-Zn, PEP3-Zn, and PEP10-Zn complexes was −127.744 ± 17.512, −104.614 ± 22.714, and −87.413 ± 21.924 kcal/mol, as shown in Table 3. The negative values indicate that the molecules have binding affinity to the target peptide. The binding energy of PEP6-Zn was higher than PEP3-Zn and PEP10-Zn.

### 2.5. Morphological Analysis and Structural Characterization of PEP and PEP-Zn

**Figure 5:**
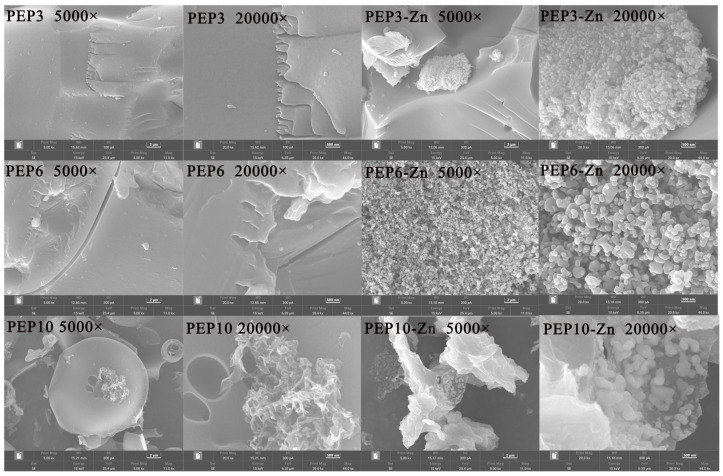
Morphological analysis and structural characterization of PEP and PEP-Zn. SEM analysis of peptides (PEP3, PEP6, PEP10) and peptide–zinc complexes (PEP3-Zn, PEP6-Zn, PEP10-Zn).

The microstructure of PEP and PEP-Zn was observed by SEM. SEM micrographs of samples in multiples of 5000 and 20,000 are shown in Figure 5. PEP3, PEP6, and PEP10 exhibited a sheet-like structure, while PEP3-Zn, PEP6-Zn, and PEP10-Zn formed a granular structure after being chelated with the zinc ion.

**Figure 6:**
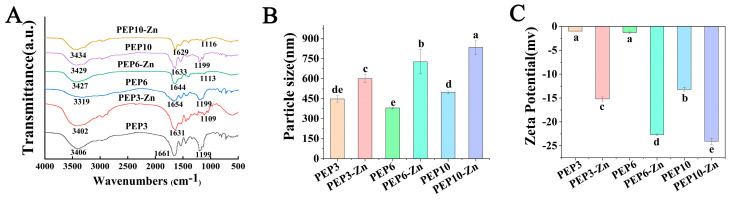
Structural characterization of PEP and PEP-Zn. FTIR spectroscopy (**A**), particle size (**B**), and zeta potential (**C**) of peptides (PEP3, PEP6, PEP10) and peptide–zinc complexes (PEP3-Zn, PEP6-Zn, PEP10-Zn). Different letters (a–e) represented the significant difference at *p* < 0.05.

We found that there were substantial differences in the infrared spectroscopy of PEP3, PEP6, PEP10 and PEP3-Zn, PEP6-Zn, PEP10-Zn (Figure 6A). The characteristic absorption peaks of PEP3, PEP6, and PEP10 appeared at 3406/3319/3429, 1661/1654/1633, and 1199/1199/1199 cm^−1^, corresponding to the stretching vibrations of hydrogen, C=O, and C–O bonds. The hydrogen bond peak of PEP3-Zn, PEP6-Zn, and PEP10-Zn shifted to 3402/3427/3434 cm^−1^ relative to the 3406/3319/3429 cm^−1^ of PEP3, PEP6, and PEP10. This indicates that zinc coordination enhances the hydrogen bonding of peptide particles [30]. Additionally, zinc coordination shifted the C=O peaks of PEP3-Zn, PEP6-Zn, and PEP10-Zn to 1631/1644/1629 cm^−1^, indicating that the zinc ion bound to the amide I band. In addition, a distinct new peak presented at 1109/1113/1116 cm^−1^ near the C-O peak in PEP3-Zn, PEP6-Zn, and PEP10-Zn, indicating that the zinc ion bound to the C-O bond to form a C-O-Zn bond [31].

The particle size distributions of PEP3, PEP6, PEP10 and PEP3-Zn, PEP6-Zn, PEP10-Zn are shown in Figure 6B. The average particle size of the PEP-Zn complexes (PEP3-Zn: 600.07 ± 29.11 nm, PEP6-Zn: 726.13 ± 91.30 nm, PEP10-Zn: 833.87 ± 51.38 nm) was higher than that of PEP (PEP3:447.17 ± 22.75 nm, PEP6: 380.27 ± 7.25 nm, PEP10: 498.10 ± 10.92 nm) (*p* < 0.05). Additionally, the polydispersity indexes (PDI) of PEP3, PEP6, PEP10 and PEP3-Zn, PEP6-Zn, PEP10-Zn were 0.442 ± 0.06, 0.510 ± 0.04, 0.626 ± 0.04 and 0.512 ± 0.02, 0.603 ± 0.03, 0.761 ± 0.03, indicating that the particles of PEP and PEP-Zn were distributed evenly. The average particle size of PEP increased due to the chelation with zinc. Meanwhile, the zeta potential of PEP and PEP-Zn suggested the stability of zinc-chelating peptides had been significantly increased when compared with PEP, as shown in Figure 6C.

### 2.6. PEP-Zn Attenuated MEHP-Induced Damage in TM3

**Figure 7:**
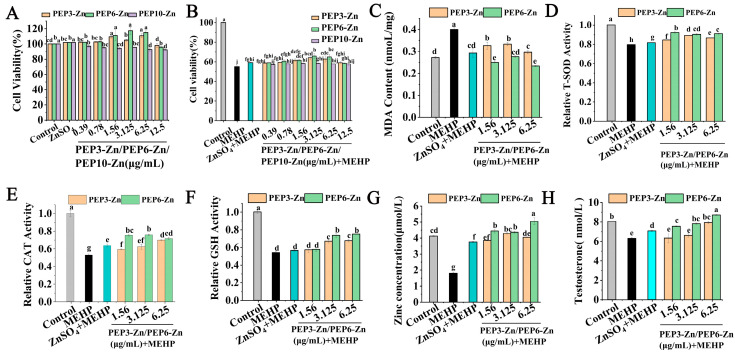
Structural PEP-Zn attenuated MEHP-induced damage in TM3. (**A**) The effect of synthetic peptide–zinc chelate on the viability of TM3 cells; (**B**) the effect of synthetic peptide–zinc chelate on the viability of MEHP-TM3 cells; (**C**) MDA level; (**D**) T-SOD level; (**E**) CAT level; (**F**) GSH level; (**G**) zinc level; (**H**) T level. Different letters (a–j) represented the significant difference at *p* < 0.05.

The cytotoxicity of PEP3-Zn, PEP6-Zn, and PEP10-Zn at concentrations of 0.39, 0.78, 1.56, 3.125, 6.25, and 12.5 µg/mL to TM3 cells was assessed by MTT assay. As shown in Figure 7A, after the treatment with PEP-Zn for 24 h, the cell viability rates were 104.94% for PEP3-Zn, 117.35% for PEP6-Zn, and 95.70% for PEP10-Zn at 3.125 µg/mL. The results indicated that PEP3-Zn, PEP6-Zn, and PEP10-Zn exhibited non-cytotoxicity towards the TM3 cells. As shown in Figure 7B, the addition of 400 μmol/L of MEHP decreased cell viability to 54.92% of the control group after incubation for 24 h. Pretreatment with PEP3-Zn, PEP6-Zn, and PEP10-Zn alleviated TM3 from MEHP-induced damage. PEP3-Zn and PEP6-Zn had better effects than PEP10-Zn, so PEP3-Zn and PEP6-Zn were chosen for the next experiment.

To further demonstrate the cytoprotective potentials of PEP3-Zn and PEP6-Zn, TM3 was preincubated with PEP3-Zn and PEP6-Zn before stimulation with MEHP. The experiment evaluated the effect of PEP3-Zn and PEP6-Zn on the oxidative stress of TM3 cells by detecting the level of MDA, and the relative activity of antioxidant enzymes (CAT, GSH, T-SOD). As shown in Figure 7C–F, compared with normal TM3 cells, the MDA content of the model group was significantly increased (*p* < 0.05), while the relative activity of antioxidant enzymes (CAT, GSH, T-SOD) was significantly decreased (*p* < 0.05). The results showed that MEHP decreased the antioxidant capacity of cells and increased the degree of oxidative stress. Compared with the model group, the significant decrease in MDA content and the significant increase in the relative activity of antioxidant enzymes (T-SOD, CAT, GSH) in the PEP6-Zn group indicated that PEP6-Zn could enhance the antioxidant capacity of TM3 cells damaged by MEHP. Additionally, PEP6-Zn had a better potential protective effect on cellular oxidative stress than PEP3-Zn. PEP3-Zn only had a protective effect at the high concentration.

The zinc level (Figure 7G) in the MEHP group was significantly lower than the control group (*p* < 0.05). Moreover, the zinc level in the ZnSO_4_, PEP3-Zn, and PEP6-Zn group significantly increased compared with the MEHP group. PEP3-Zn and PEP6-Zn increased the zinc concentration more effectively than ZnSO_4_. Moreover, testosterone is mainly synthesized by TM3 cells, which plays an important role in maintaining male sperm production [32,33]. As shown in Figure 7H, the testosterone content of the control group was 8.05 ± 0.029 nmol/L. Treatment with 400 μmol/L of MEHP markedly reduced the testosterone content of the TM3 cells (6.29 ± 0.112 nmol/L). PEP6-Zn increased testosterone content more effectively than PEP3-Zn at the concentrations of 1.56, 3.125, and 6.25 μg/mL (*p* < 0.05).

### 2.7. PEP6-Zn Alleviate the MEHP-Mediated TM3 Cell Apoptosis

**Figure 8:**
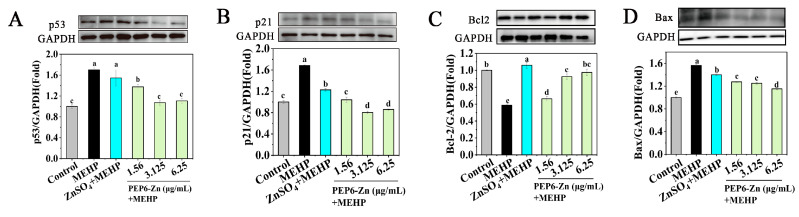
The protein levels of apoptotic markers (**A**–**D**) after PEP6-Zn and MEHP exposure (n = 3). The results were expressed as the means ± standard deviations. Different letters (a–d) represented the significant difference at *p* < 0.05.

Research has found that DEHP induced apoptosis in TM3 cells by upregulating p53 expression, the knockout of the p53 gene significantly reduces DEHP-induced apoptosis, while the overexpression of p53 significantly induces apoptosis in TM3 cells [34]. In order to further study the mechanism of PEP6-Zn on apoptosis induced by MEHP in TM3 cells, we used Western blot analysis to detect the protein levels of apoptosis-related markers. The protein levels of p53, p21, and Bax in the MEHP group were significantly increased, while the protein level of Bcl-2 was significantly decreased. Compared with the MEHP group, the protein level of p53 and p21 and Bax in the MEHP + PEP6-Zn group decreased significantly (Figure 8A,B,D), and the protein level of Bcl-2 was increased (Figure 8C). These results indicated that PEP6-Zn can reduce the protein levels of apoptosis markers caused by MEHP in TM3 cells. In comparison, the effect of PEP6-Zn treatment was better than ZnSO_4_.

**Figure 9:**
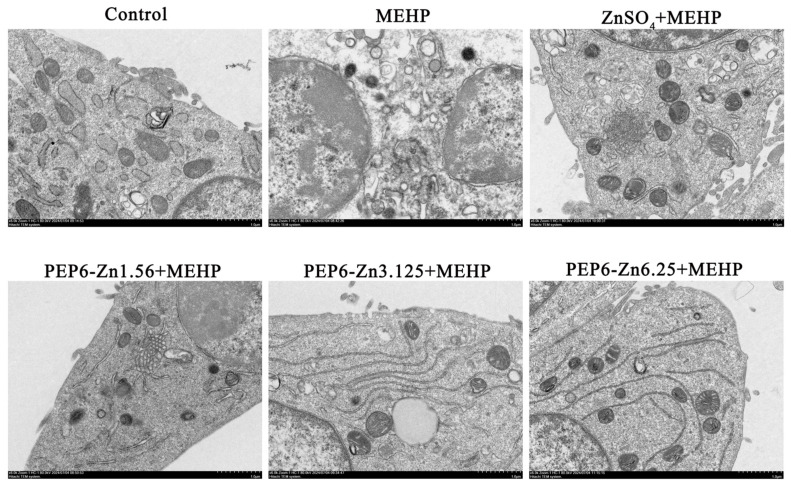
Observation of the effect of PEP6-Zn on the ultrastructural changes of MEHP-induced TM3 cells through transmission electron microscopy (scale bar: 1 μm).

In this experiment, the apoptotic morphological changes of TM3 cells caused by MEHP were observed by transmission electron microscopy (Figure 9). Compared with the control group, after MEHP treatment, the cytoplasm of TM3 cells showed vacuoles, some mitochondria were swollen, the chromatin of the nucleus was condensed, and typical apoptotic vesicles were also presented. After different concentrations of PEP6-Zn treatment, the ultrastructure of TM3 cells was damaged, but all of them were better than the MEHP group. The ZnSO_4_ pretreatment group also alleviated the damage of MEHP on TM3 cells.

## 3. Discussion

Di(2-ethylhexyl) phthalate (DEHP) is a common environmental endocrine disruptor that is widely used in plastic products worldwide and it poses major risks to male reproduction. In in vivo experiments, our previous research had found that the oyster peptide–zinc complex alleviates the reproductive damage caused by DEHP in mice by restoring testicular zinc homeostasis [35]. The aim of this study was to develop and characterize novel ethanol-soluble oyster zinc-binding peptides and to investigate the protective mechanisms of the peptide–zinc complex on MEHP-induced TM3 cells in in vitro experiments.

In the present study, A3 was isolated from the OP-ES using the IMAC method, and the fractions containing different peptide sequences were evaluated with the aim of studying their structures and bioactivities. Immobilized metal ion affinity chromatography (IMAC) is a widely used separation method for purifying metal-binding peptides. It is based on the specific interaction between certain amino acid side chains exposed on the surface of proteins (mainly His). In this research, the content of histidine from A1, A2, and A3 was 0.66%, 25.10%, and 30.50%. The results in this paper are consistent with those of Peng [36]. High concentrations of histidine have also been reported in other studies of food peptide isolates using IMAC, including the tilapia skin collagen peptide and chickpea protein [16,37]. The amino acid composition of a peptide is the most basic indicator of its biological activity. Zn^2+^ binds more favorably to His-rich peptides, and His is also a good transition-metal binder and promotes the solubility of Zn^2+^ [38]. Additionally, the cell viability of the MEHP-injured group decreased to 69.60 ± 0.14% (*p* < 0.05), the cell viability of the A1Z, A2Z, and A3Z pretreatment groups was 71.15 ± 0.92%, 75.30 ± 1.38%, and 78.06 ± 0.67%, respectively, and A3Z had the best protective effect on MEHP-injured TM3 cells. From the results above, it can be seen that A3Z could inhibit the MEHP-induced oxidative stress damage in TM3 cells by increasing the enzymatic antioxidant system activity and decreasing the MDA content. The testosterone levels in culture supernatants were decreased when the Leydig cell was treated with MEHP, and finally increased apoptosis [39]. The current results show that the testosterone levels in the MEHP group were significantly lower than those of the controls, and A3Z increased by 57.67% compared with the control group, which was consistent with previously reported results [40]. Among the results above, when administered at the same concentration or at a low concentration, A3Z showed stronger antioxidant activity and better effect.

In silico analysis has been widely applied to develop a wide range of bioactive metal-chelating peptides. In this research, 12 peptides were identified and selected for their good solubility and stability, and none of them were toxic from A3. Among the 12 zinc-chelating peptides, it can be concluded that each peptide contained one or more amino such as His, Glu, Asp, and Ser [41]. PEP3, PEP6, PEP9, PEP10, and PEP11 have high docking energy, which demonstrated good chelation of peptides with Zn^2+^. To evaluate the potential of the five peptides as metal-chelating agents, their chelating ability for Zn^2+^ was determined. The peptides of EHAPNHDNPGDL (PEP6), GHPGLPGDAGPEGPR (PEP3), and HLDDILFS (PEP10) had the highest zinc-binding activity among the five synthetic peptides. EHAPNHDNPGDL (PEP6) exhibited the highest zinc-binding activity and was higher than that of the OP-ES (27.50 ± 0.41%).

The interaction between proteins and metal surfaces is widely present in biomedical applications, such as wound therapy and drug delivery [42]. Molecular dynamics simulations combine the advantages of accuracy and computational feasibility and are widely used to describe the interactions between metal element surfaces and proteins or peptides [43,44]. The docking conformation of PEP6-Zn suggested that the dodecapeptide formed five binding bonds (Asn-5, Asp-7, Asn-8, His-2, and Asp-11) with zinc. This indicated that PEP6-Zn has a tight affinity for zinc. A previous study had discovered that gold surfaces can induce conformational changes in collagen molecules, leading to alterations in their secondary and tertiary structures, which can affect the mechanical properties and biological functions of collagen, such as cell adhesion, proliferation, and differentiation [45]. In accordance with a previous study, compared with ferrous sulfate, FEDPEFE-ferrous chelate showed more stability in salt solution and simulated gastrointestinal juice, and the heptapeptide (FEDPEFE) forms six binding bonds, with ferrous irons [46]. Molecular dynamics simulations provide a new perspective to understand the adsorption of oyster peptides and zinc, and guide the development of biomaterials based on oyster peptides chelating zinc.

Cellular zinc homeostasis is the maintenance of zinc ion balance within cells, which is crucial for the normal physiological functions of cells [47]. MEHP caused a decrease in zinc content in the TM3 cells and disrupted the intracellur zinc homeostasis. The EHAPNHDNPGDL-Zn group had a higher zinc level than the PEP3-Zn group and ZnSO_4_ group, and it regulated the zinc homeostasis and maintained a relatively constant intracellular zinc level at a low concentration. A previous study has found that the mung bean protein peptide SSEDQPFNLR-zinc chelate promoted zinc uptake and significantly increased zinc ion transport in the cell model [48]. Our previous study revealed that exposure to DEHP decreased the concentrations of zinc (Zn) in mouse testis, and the oyster peptide–zinc complex significantly improved the homeostasis of the zinc element within the testis tissues [35]. The organic zinc of EHAPNHDNPGDL-Zn had a better effect on restoring zinc content than inorganic zinc sulfate. Organic zinc complexes are believed to deliver micronutrients to the intestine in a form that is readily bioaccessible and available to the intestinal epithelium, which can prevent the formation of insoluble metal complexes during gastrointestinal digestion [49]. Available evidence suggests that zinc deficiency promotes testicular cell apoptosis in mice, and the pharmacological doses of zinc increase testosterone production [50,51]. By recognizing the importance of TM3 cells in testosterone synthesis, we subsequently investigated the effect of zinc on testosterone secretion from MEHP-TM3 cells. As expected, ZnSO_4_, PEP3-Zn, and PEP6-Zn treatment effectively restored the diminished testosterone levels caused by MEHP treatment. Previous studies have shown that zinc pretreatment reduced sertoli cell toxicity caused by ethanol, and also restored the TM3 cell damage caused by heat stress [40,52]. In addition, PEP6-Zn had a better potential protective effect on cellular oxidative stress and higher testosterone content and zinc content than PEP3-Zn.

Zhao et al. have found that after MEHP treatment, the TM3 cell apoptosis cells increased significantly, which confirmed the adverse effect of MEHP on Leydig cells [39]. However, the mechanism is not completely clear. In this research, the protein levels of p53, p21 and Bax in the MEHP group were significantly increased, while the protein level of p53, p21, and Bax in the MEHP + PEP6-Zn group decreased significantly, and the protein level of Bcl-2 increased. Our results are consistent with previous studies indicating that the peony seed protein hydrolysate alleviates the terfenadine-induced apoptosis of zebrafish embryos by inhibiting the expression of p53 [53]. And zinc pretreatment restored morphine-induced apoptosis as well as p53 overexpression [54]. The occurrence of apoptosis is regulated by many genes, mainly the caspase and Bcl-2 families [55]. p53 can regulate apoptosis through the mitochondrial pathway by targeting the Bcl2 family of proteins [56,57]. Using transmission electron microscopy, a previous study had observed that the mitochondrial morphology was abnormal with fewer mitochondrial cristae and denser outer membranes in Leydig cells after MEHP exposure [58]. After different concentrations of PEP6-Zn treatment, the ultrastructure of TM3 cells was damaged, but all of them were better than the MEHP group.

In conclusion, EHAPNHDNPGDL-Zn could increase the testosterone of TM3 cells damaged by MEHP. Meanwhile, we showed that the oxidative stress could induce the apoptosis of TM3 cells, accompanied with the increased protein levels of p53, p21, and Bax, and decreased protein levels of Bcl-2, while PEP6-Zn could inhibit the oxidative stress dramatically and alleviate MEHP-induced apoptosis. These findings can be used as a theoretical basis for the development of new peptide–zinc supplements.

## 4. Materials and Methods

### 4.1. Materials and Chemicals

The Crude Oyster peptides (molecular weight: between 180–1000 Da) were provided by Hainan Shengmeinuo Biotechnology Co., Ltd., and ZnSO_4_.7H_2_O and MEHP were obtained from Sigma Aldrich (Z0251/796832, Sigma, MD, USA). Chelax-100 was purchased from Bio-Rad (Shanghai, China). All other chemicals and reagents used in this study were of analytical grade.

### 4.2. Enrichment of Zinc-Chelating Peptides Using Immobilised Metal Affinity Chromatography

The preparation of the IMAC-Zn^2+^ column was carried out according to the method of Matsumoto [59] and the product specification. The IMAC column (16 mm × 40 mm, China) was cleaned and fixed vertically on a wire-frame stage, and the IMAC Sepharose™ 6 Fast Flow (50 mL, 17092108, GE Healthcare, Waukesha, WI, USA) was slowly introduced into the column along a glass rod. The packing was water-sealed with ultrapure water and kept wet. Ten times the column volume of ultrapure water was used to elute ethanol from the IMAC packings, followed by loading the IMAC packings with five times the volume of 0.2 M ZnCl_2_ (pH = 5) to allow Zn^2+^ to fully bind to the IMAC packings, and then five times the volume of equilibrium buffer (20 mM PBS, pH = 7.4) to elute the unbound Zn^2+^. The solution (10 mg/mL) was prepared by the OP-ES in equilibrium buffer, filtered through a 0.22 μm aqueous microporous filter membrane, and the OP-ES was uploaded in 5 mL. The OP-ES was finally obtained using the previous method [22,35]. The unbound peptides were first removed by equilibrium buffer (20 mM PBS, pH = 7.4) (A1), and the weakly and strongly bound peptides were successively collected with elution buffer (20 mM PBS, pH = 4.0) (A2, A3), and then the bound peptides were treated with Chelex resin [60].

### 4.3. Analysis of Amino Acid Composition

A1, A2, and A3 (100 mg) were hydrolyzed and an amino acid analyzer (A300, MembraPure, Berlin, Germany) was used to determine the amino acid composition of A1, A2, and A3.

### 4.4. MALDI-TOF/MS Analysis

The exact molecular weights of the isolated and purified peptides were characterized by MALDI-TOF-MS (Ultraflextreme, Bruker, Germany).

### 4.5. Preparation of Peptide–Zinc Chelates

The lyophilized powers of A1, A2, and A3 were added to deionized water (7.5 mg/mL), and ZnSO_4_.7H_2_O (20 mmol/L) was then added at a proportion of (2:1). The pH of the reaction solution was adjusted to 6.5 and maintained in a water bath at 40 degrees Celsius for 1 h, then it was centrifuged at a 11,740× *g* force and the precipitate was removed. The supernatant and anhydrous ethanol were mixed thoroughly (*v*:*v* = 1:3), centrifuged at 11,740× *g* force for 10 min, and then the precipitate was collected for freeze-drying [61].

### 4.6. Effect of Isolated and Purified Peptides and Their Zinc Chelates in MEHP-TM3 Cells

The immature TM3 Leydig cells used in the study were purchased from the Cell Bank of Type Culture Collection of the Chinese Academy of Sciences (GNM24, Shanghai, China), which was established in 1980 by Mather JP [62]. TM3 is a commonly used Leydig cell line that is extracted from immature mouse testes at an age of 11–13 days. The cells were maintained in Dulbecco’s Modified Eagle Medium. After they reached a confluence of 70%, TM3 was then treated with 50, 100, 200, 400, 800 μg/mL of A1, A2, A3, and 3.125, 6.25, 12.5, 25, 50, 100, 200 μg/mL of A1Z, A2Z, A3Z for 24 h. Cell viability was determined by MTT assay.

The protective effect of A1, A2, A3 and A1Z, A2Z, and A3Z was evaluated by establishing MEHP (400 µM)-induced oxidative stress [2]. The TM3 cells were pretreated with 200, 400, 800 μg/mL of A1, A2, A3, and 12.5, 25, 50 μg/mL of A1Z, A2Z, A3Z for 24 h. Then the treatment time of MEHP was 24 h. Cell viability was determined by MTT assay. Oxidative stress indices (GSH, T-SOD, MDA) were detected by using commercial kits, and the test was carried out by referring to the instructions of the kits (Nanjing, China). Testosterone levels were detected by the ELISA kit (MEIMIAN, Yancheng, China).

### 4.7. Identification of Zinc-Binding Peptides via HPLC-MS/MS

The peptide sequence of A3 isolated and purified from IMAC was analyzed by Bio-Tech Pack Technology Company Ltd. (Beijing, China). The HPLC column was an Acclaim PepMap RPLC C18 (150 μm × 150 mm, 1.9 μm). The mobile phase was (A) 0.1% formic acid; B was 0.1% formic acid and 80% ACN; the elution procedure was as follows: 0–3 min, 4–8% B; 3–89 min, 8–28% B; 89–109 min, 28–40% B; 109–110 min, 40–95% B; and 110–120 min, 95% B.

### 4.8. In Silico Investigation of Identified Peptides and Molecular Docking

The ToxinPred server was used to analyze the toxicity of the peptides and is available at https://webs.iiitd.edu.in/raghava/toxinpred/design.php (accessed on 28 March 2024). Peptide solubility was assessed by the online Innovagen server at http://www.innovagen.com/proteomics-tools (accessed on 28 March 2024). The instability index of the peptides was predicted using the ProtParam tool at https://web.expasy.org/protparam/ (accessed on 28 March 2024).

The molecular docking of the peptides and Zn^2+^ was conducted by the method reported by Du [63], with slight modifications. First, the structures of the peptides were performed by Rosetta 3.14 [64]. Second, the structure of the zinc ion was obtained from PubChem. Molecular docking analysis was conducted as previously described [65,66] using the Discovery Studio (version 2.5, Dassault Systems Bioviato, San Diego, CA, USA), and then the binding energy was calculated and potential interactions were assessed.

### 4.9. Synthesis of Peptides and Evaluation of Zinc-Chelating Ability

The five selected sequences were synthesized by Qiangyao Biotechnology Co., Ltd. (Shanghai, China) with purity higher than 96%. These synthetic peptides were assessed for zinc-chelating ability using the methods below.

The determination of zinc-binding ability was measured according to the method described by Li et al., with some modifications [63,67]. The total amount of the Zn^2+^: 3 mL aliquot of peptide-chelated zinc solution was made up to 12 mL with deionized water, and the zinc content was assayed by ICP-OES as M0. The amount of chelating zinc was as follows: three milliliters of the peptide–zinc chelate solution was centrifuged for 15 min (11,740× *g* force), and the supernatant was mixed with 3× anhydrous ethanol for 2 h and centrifuged to obtain the final precipitate, which was finally fixed to 12 mL with distilled water, and the zinc content was assayed by ICP-OES as M1. The zinc-chelating ability was calculated using the following Equation (1):

(1) $$\mathrm{The}\;\mathrm{amount}\;\mathrm{of}\;\text{zinc-chelating}\;\mathrm{ability}\;(\%)=\frac{M1}{M0}$$

where M0 and M1 were the total amount of zinc and the amount of chelating zinc, respectively.

### 4.10. Molecular Dynamics Simulation

To validate the docking results, a 200 ns MD simulation was conducted with a peptide and zinc ion. The MD simulation was performed using the GROMACS program (version 2020) and the AMBER99SB-ILDN force field, following a previously reported protocol [68]. The binding free energy between the peptide and zinc ion was calculated using the molecular mechanics/Poisson−Boltzmann surface area (MM/PBSA) method [28].

### 4.11. Physicochemical Characterization of Peptide and Peptide–Zinc Complex

The PEP3-Zn/PEP6-Zn/PEP10-Zn complex was prepared using the method of 4.5 above. PEP and PEP-Zn were evaluated by scanning electron microscopy (DSM 940A, ZEISS, Jena, Germany), fourier transform infrared spectroscopy (Bruker, Etlingen, Germany), and the Zeta sizer Nano ZS-90 instrument (Malvern Instruments, Malvern, UK).

### 4.12. Effect of Synthetic Peptides and Their Zinc Chelates in MEHP-TM3 Cells

The toxicity of the synthetic peptides chelated with Zn was measured by the MTT assay using TM3. The protective effect of PEP3-Zn, PEP6-Zn, and PEP10-Zn was evaluated by establishing MEHP (400 µM)-induced oxidative stress. The experiment was divided into six groups, including the control group, model group (MEHP solution), positive drug group with 10 μM zinc (zinc sulfate, Sigma, USA), and protection group treated with different concentrations (0.39, 0.78, 1.56, 3.125, 6.25, 12.5 µg/mL of PEP3-Zn/PEP6-Zn/PEP10-Zn). After 24 h, PEP-Zn was aspirated and exposed to MEHP for 24 h. Cell viability was determined by MTT assay. The experimental methods of oxidative stress indices (MDA, T-SOD, CAT, GSH) and testosterone levels were consistent with the method above. The Elabscience zinc ion detection kit (E-BC-K137-M, Elabscience, Wuhan, China) was used to measure the intracellular zinc ion concentration in TM3 cells.

### 4.13. Western Blotting

Western blot analysis was performed following previously reported procedures [69]. The anti-p53 (ab131442), anti p21 (EPR18021), anti-Bcl2 (EPR17509), and anti-Bax (E63) antibodies were purchased from Abcam (Cambridge, MA, USA). GAPDH (14C10, CST) was used as a loading control. The densitometry analysis was calculated using ImageJ software 1.53q (National Institutes of Health, Bethesda, MD, USA).

### 4.14. Transmission Electron Microscopy Analysis

TM3 cells were cultured into 10 cm dishes, and the cells were cultured and administered by the method of 4.12. The cells were washed three times with PBS at the end of the culture and centrifuged at 3500× *g* for 10 min, the supernatant was discarded, and 2.5% glutaraldehyde was added slowly to avoid dispersing the cell mass, and stored at 4 °C. The samples were then observed with a transmission electron microscope (Hitachi HT7800) after pretreatment.

### 4.15. Statistical Analysis

The results were expressed as the mean ± standard deviation (SD). Significance was analyzed by univariate by one-way analysis variance in IBM SPSS Statistics 26 software, followed by Tukey’s test. Values of *p* < 0.05 were considered significant.

## Figures and Tables

**Table 1:** Sequence of peptides identified by LC-MS/MS from A3.

No	Peptide Sequence (a)	Molecular Mass (Da)	Instability Index	Toxicity	Estimated Solubility
PEP1	SETGAGKHVPR	1138.59	14.1	Non-Toxin	good
PEP2	KQVHPDTGVSSKAM	1500.74	10.33	Non-Toxin	good
PEP3	GHPGLPGDAGPEGPR	1413.68	26.81	Non-Toxin	good
PEP4	KQVHPDTGVSSK	1282.67	10.38	Non-Toxin	good
PEP5	YHPTKPGDYT	1178.55	2.43	Non-Toxin	good
PEP6	EHAPNHDNPGDL	1315.56	16.53	Non-Toxin	good
PEP7	SRLPGQCEMKH	1358.63	37.25	Non-Toxin	good
PEP8	AIGDHDGHVGL	1090.52	1.37	Non-Toxin	good
PEP9	DVHPEHPY	993.44	13.85	Non-Toxin	good
PEP10	HLDDILFS	959.49	32.83	Non-Toxin	good
PEP11	YHDHDVPCA	1113.44	35.62	Non-Toxin	good
PEP12	DYTKHPSKPD	1187.57	1.81	Non-Toxin	good

(a) Amino acids are represented by letter codes: A: Ala; C: Cys; D: Asp; E: Glu; F: Phe; G: Gly; H: His; I: Ile; K: Lys; L: Leu; M: Met; N: Asn; P: Pro; Q: Gln; R: Arg; S: Ser; T: Tre; V: Val; Y: Tyr.

**Table 2:** Molecular docking of peptides with zinc from A3 identified by LC-MS/MS.

No	Peptide Sequence	Chelating Sites	Docking Energy (kj/mol)	Types	Distance (Å)
PEP1	SETGAGKHVPR	Glu-2	18.4682	C-C	2.2
PEP2	KQVHPDTGVSSKAM	His-4	25.3241	M-A; P-C	3.6; 1.9
		Asp-6		M-A; C-C	2.3; 2.4
PEP3	GHPGLPGDAGPEGPR	Asp-8	29.7068	M-A	3.0
		His-2		C-C	3.9
		Gly-13		M-A	2.9
PEP4	KQVHPDTGVSSK	His-4	21.8462	P-C	2.1
		Asp-6		C-C	2.1
PEP5	YHPTKPGDYT	Tyr-1	13.7164	M-A	1.1
		Asp-8		C-C	1.3
PEP6	EHAPNHDNPGDL	Asn-5	32.2966	M-A;	2.2
		Asp-7		M-A; C-C	2.2; 2.7
		Asn-8		M-A	2.2
		Asp-11		C-C	1.9
PEP7	SRLPGQCEMKH	Gln-6	16.8824	M-A	2.6
		His-11		C-C	1.9
PEP8	AIGDHDGHVGL	Asp-4	19.9915	C-C	1.9
		Asp-6		M-A; C-C	2.0; 2.8
PEP9	DVHPEHPY	Asp-1	28.5144	C-C; M-A	2.3; 2.1
		His-3		P-C	1.9
		His-6		P-C	1.9
PEP10	HLDDILFS	His-1	26.6488	P-C	3.2
		Asp-3		M-A; C-C	2.3; 3.2
		Asp-4		M-A; C-C	2.3; 2.8
PEP11	YHDHDVPCA	ASP-3	28.1060	M-A	1.8
		His-4		P-C	1.7
PEP12	DYTKHPSKPD	Asp-1	14.4421	C-C	2.9

Note: The abbreviations of the types are as follows: M-A: metal–acceptor; C-C: charge–charge; P-C: pi–cation.

**Table 3:** Energy components of PEP and zinc complexes (kJ/mol).

Type	PEP6-Zn	PEP3-Zn	PEP10-Zn
*E_VDW_*	123.431 ± 14.199	73.546 ± 13.435	62.876 ± 10.164
*E_ELE_*	−2026.575 ± 39.846	−1085.907 ± 33.538	−1185.357 ± 83.788
*E_GB_*	1777.041 ± 53.483	909.404 ± 40.165	1036.67 ± 98.489
*E_SA_*	−1.641 ± 0.05	−1.657 ± 0.052	−1.602 ± 0.105
*G_binding energy_*	−127.744 ± 17.512	−104.614 ± 22.714	−87.413 ± 21.924

Note: *E_VDW_*, van der Waals energy. *E_ELE_*, electrostatic energy. *E_GB_*, polar contribution to solvation. *E_SA_*, non-polar contribution to solvation. *G_binding energy_*, binding free energy.

## Data Availability

Data are available upon request.

## References

[1] Martínez M.A., Rovira J., Prasad Sharma R., Nadal M., Schuhmacher M., Kumar V. (2018). Comparing dietary and non-dietary source contribution of BPA and DEHP to prenatal exposure: A Catalonia (Spain) case study. Environ. Res..

[2] Hong Y., Zhou Y., Shen L., Wei Y., Long C., Fu Y., Wu H., Wang J., Wu Y., Wu S. (2021). Exposure to DEHP induces testis toxicity and injury through the ROS/mTOR/NLRP3 signaling pathway in immature rats. Ecotoxicol. Environ. Saf..

[3] Zhang Q., Sun Y., Zhang Q. (2020). Phthalate exposure in Chinese homes and its association with household consumer products. Sci. Total Environ..

[4] Di Bella G., Saitta M., Lo Curto S., Salvo F., Licandro G., Dugo G. (2001). Production process contamination of citrus essential oils by plastic materials. J. Agric. Food Chem..

[5] Xu J., Wang L., Zhang L., Zheng F., Wang F., Leng J., Wang K., Héroux P., Shen H.M., Wu Y. (2021). Mono-2-ethylhexyl phthalate drives progression of PINK1- parkin-mediated mitophagy via increasing mitochondrial ROS to exacerbate. Redox Biol..

[6] Beko G., Weschler C.J., Langer S. (2013). Children’s phthalate intakes and resultant cumulative exposures estimated from urine compared with estimates from dust ingestion, inhalation and dermal absorption in their homes and daycare centers. PLoS ONE.

[7] Fong J.P., Lee F.J., Lu I.S., Uang S.N., Lee C.C. (2014). Estimating the contribution of inhalation exposure to di-2-ethylhexyl phthalate (DEHP) for PVC production workers, using personal air sampling and urinary metabolite monitoring. Int. J. Hyg. Environ. Health.

[8] Wei Y., Zhou Y., Tang X.L., Liu B., Shen L.J., Long C., Lin T., He D., Wu S., Wei G. (2018). Testicular developmental impairment caused by flutamide-induced and DEHPinduced cryptorchid rat models is mediated by excessive apoptosis and deficient autophagy. Toxicol. Mech. Methods.

[9] Qin Y., Zhang J., Avellan-Llaguno R.D., Zhang X., Huang Q. (2021). DEHP-elicited small extracellular vesicles miR-26a-5p promoted metastasis in nearby normal A549 cells. Environ. Pollut..

[10] Wang J., Zhao T., Chen J., Kang L., Wei Y., Wu Y., Han L., Shen L., Long C., Wu S. (2021). Multiple transcriptomic profiling: p53 signaling pathway is involved in DEHP-induced prepubertal testicular injury via promoting cell apoptosis and inhibiting cell proliferation of Leydig cells. J. Hazard. Mater..

[11] Park J.D., Habeebu S.S., Klaassen C.D. (2020). Testicular toxicity of di-(2-ethylhexyl)phthalate in young Sprague-Dawley rats. Toxicology.

[12] Gao H.T., Di Q.N., Qian L.L., Lu L., Li R.X., Cao W.X., Xu Q. (2020). Zinc supplement ameliorates phthalates-induced reproductive toxicity in male rats. Chemosphere.

[13] Stehbens W.E. (2003). Oxidative stress, toxic hepatitis, and antioxidants with particular emphasis on zinc. Exp. Mol. Pathol..

[14] Joshua Ashaolu T., Lee C.C., Opeolu Ashaolu J., Pourjafar H., Jafari S.M. (2023). Metal-binding peptides and their potential to enhance the absorption and bioavailability of minerals. Food Chem..

[15] Syam A., Sari N.P., Thaha A.R., Jafar N., Salam A., Mallongi A. (2020). The effect of pumpkin seed flour (*Cucurbita moschata* Durch) on zinc serum levels in malnourished Wistar rats. Enferm. Clin..

[16] Katimba H.A., Wang R., Cheng C. (2023). Current findings support the potential use of bioactive peptides in enhancing zinc absorption in humans. Crit. Rev. Food Sci. Nutr..

[17] Li J., Gong C., Wang Z., Gao R., Ren J., Zhou X., Wang H., Xu H., Xiao F., Cao Y. (2019). Oyster-Derived Zinc-Binding Peptide Modified by Plastein Reaction via Zinc Chelation Promotes the Intestinal Absorption of Zinc. Mar. Drugs.

[18] Feng Y., Jiang S., Wang Z., Li S., Zeng M. (2020). Oyster hydrolysate-zinc complex ameliorates carrageenan-induced rat prostatitis via an anti-inflammatory mechanism and reduced oxidative stress. J. Funct. Foods.

[19] Wu S., Zhu Z., Chen M., Huang A., Xie Y., Hu H., Zhang J., Wu Q., Wang J., Ding Y. (2023). Comparison of Neuroprotection and Regulating Properties on Gut Microbiota between Selenopeptide Val-Pro-Arg-Lys-Leu-SeMet and Its Native Peptide Val-Pro-Arg-Lys-Leu-Met In Vitro and In Vivo. J. Agric. Food Chem..

[20] Chen D., Mu X., Huang H., Nie R., Liu Z., Zeng M. (2014). Isolation of a calcium-binding peptide from tilapia scale protein hydrolysate and its calcium bioavailability in rats. J. Funct. Foods.

[21] He W., Su G., Sun-Waterhouse D., Waterhouse G.I.N., Zhao M., Liu Y. (2019). In vivo anti-hyperuricemic and xanthine oxidase inhibitory properties of tuna protein hydrolysates and its isolated fractions. Food Chem..

[22] Yuan Z., Yang M., Zhu D., Wu D., Cheng S., Wu C. (2023). Immunomodulatory effect of ethanol-soluble polypeptides from atlantic cod (gadus morhua). Food Sci. Hum. Wellness.

[23] Kong X.Z., Bao S.S., Song W.G., Hua Y.F., Zhang C.M., Chen Y.M., Li X.F. (2021). Contributions of ethanol fractionation on the properties of vegetable protein hydrolysates and differences in the characteristics of metal (ca, zn, fe)-chelating peptides. LWT-Food Sci. Technol..

[24] Wu H., Liu Z., Zhao Y. (2012). Enzymatic preparation and characterization of iron-chelating peptides from anchovy (Engraulis japonicus) muscle protein. Food Res. Int..

[25] Xu Z.L., Hu Q.H., Xie M.H., Liu J.H., Su A.X., Xu H., Yang W.J. (2023). Protective effects of peptide KSPLY derived from Hericium erinaceus on H_2_O_2_-induced oxidative damage in HepG2 cells. Food Sci. Hum. Wellness.

[26] Zhang T.D., Ma Y.B., Li H.C., Chong T., Wang Z.M., Zhang L.D. (2020). Low Dose of Genistein Alleviates Mono-(2-Ethylhexyl) Phthalate-Induced Fetal Testis Disorder Based on Organ Culture Model. Oxidative Med. Cell. Longev..

[27] Tu M., Liu H., Zhang R., Chen H., Mao F., Cheng S., Lu W., Du M. (2018). Analysis and Evaluation of the Inhibitory Mechanism of a Novel Angiotensin-I-Converting Enzyme Inhibitory Peptide Derived from Casein Hydrolysate. J. Agric. Food Chem..

[28] Wang X., Deng Y., Zhang Y., Zhang C., Liu L., Liu Y., Jiang J., Xie P., Huang L. (2023). Screening and Evaluation of Novel α-Glucosidase Inhibitory Peptides from Ginkgo biloba Seed Cake Based on Molecular Docking Combined with Molecular Dynamics Simulation. J. Agric. Food Chem..

[29] Ali S.A., Hassan M.I., Islam A., Ahmad F. (2014). A review of methods available to estimate solvent-accessible surface areas of soluble proteins in the folded and unfolded states. Curr. Protein Pept. Sci..

[30] Xia Q., Liang Y., Cao A. (2023). Preparation and characterization of pH-responsive metal-polyphenol structure coated nanoparticles. Food Sci. Hum. Wellness.

[31] Zhou S.P., Zhang J.P., Yin X.Y., Xiong C.Y., Zhang N., Gao Z.Y., Fan J.F., Zhang W.W., Wang J.T. (2024). Achieving high-performance peptide-based foam via moderate hydrolysis and zinc coordination of oat proteins. Food Hydrocoll..

[32] Shima Y., Miyabayashi K., Haraguchi S., Arakawa T., Otake H., Baba T., Matsuzaki S., Shishido Y., Akiyama H., Tachibana T. (2013). Contribution of Leydig and Sertoli cells to testosterone production in mouse fetal testes. Mol. Endocrinol..

[33] Zhou R., Wu J., Liu B., Jiang Y., Chen W., Li J., He Q., He Z. (2019). The roles and mechanisms of Leydig cells and myoid cells in regulating spermatogenesis. Cell. Mol. Life Sci..

[34] Li Y., Xu L., Hao C., Yang S., Wang J., Chen J. (2024). ARTS is essential for di-2-ethylhexyl phthalate (DEHP)-induced apoptosis of mouse Leydig cells. Ecotoxicol. Environ. Saf..

[35] Lu Z., Huang Q., Chen F., Li E., Lin H., Qin X. (2023). Oyster Peptide-Zinc Complex Ameliorates Di-(2-ethylhexyl) Phthalate-Induced Testis Injury in Male Mice and Improving Gut Microbiota. Foods.

[36] Peng M., Lu D., Yu M., Jiang B., Chen J. (2022). Identification of zinc-chelating pumpkin seed (*Cucurbita pepo* L.) peptides and in vitro transport of peptide-zinc chelates. J. Food Sci..

[37] Meng K., Chen L., Xia G., Shen X. (2021). Effects of zinc sulfate and zinc lactate on the properties of tilapia (*Oreochromis niloticus*) skin collagen peptide chelate zinc. Food Chem..

[38] Wang C., Li B., Ao J. (2012). Separation and identification of zinc-chelating peptides from sesame protein hydrolysate using IMAC-Zn^2+^ and LC-MS/MS. Food Chem..

[39] Zhao T., Wang J., Wu Y., Han L., Chen J., Wei Y., Shen L., Long C., Wu S., Wei G. (2021). Increased m6A modification of RNA methylation related to the inhibition of demethylase FTO contributes to MEHP-induced Leydig cell injury. Environ. Pollut..

[40] Xiong Y., Li J., He S. (2022). Zinc Protects against Heat Stress-Induced Apoptosis via the Inhibition of Endoplasmic Reticulum Stress in TM3 Leydig Cells. Biol. Trace Elem. Res..

[41] Li S.T., Hu X., Li L.L., Yang X.Q., Chen S.J., Wu Y.Y., Yang S.L. (2021). Preparation, purification and identification of iron-chelating peptides derived from tilapia (*Oreochromis niloticus*) skin collagen and characterization of the peptide-iron complexes. LWT–Food Sci. Technol..

[42] Pei P., Chen L., Fan R., Zhou X.R., Feng S., Liu H., Guo Q., Yin H., Zhang Q., Sun F. (2022). Computer-Aided Design of Lasso-like Self-Assembling Anticancer Peptides with Multiple Functions for Targeted Self-Delivery and Cancer Treatments. ACS Nano.

[43] Penna M.J., Mijajlovic M., Biggs M.J. (2014). Molecular-level understanding of protein adsorption at the interface between water and a strongly interacting uncharged solid surface. J. Am. Chem. Soc..

[44] Bellucci L., Bussi G., Di Felice R., Corni S. (2017). Fibrillation-prone conformations of the amyloid-beta-42 peptide at the gold/water interface. Nanoscale.

[45] Deguchi S., Yokoyama R., Maki T., Tomita K., Osugi R., Hakamada M., Mabuchi M. (2021). A new mechanism for reduced cell adhesion: Adsorption dynamics of collagen on a nanoporous gold surface. Mater. Sci. Eng. C Mater. Biol. Appl..

[46] Yan X., Yue Y., Guo B., Zhang S., Ji C., Chen Y., Dai Y., Dong L., Zhu B., Lin X. (2024). Novel microbial fermentation for the preparation of iron-chelating scallop skirts peptides-its profile, identification, and possible binding mode. Food Chem..

[47] Chen B., Yu P., Chan W.N., Xie F., Zhang Y., Liang L., Leung K.T., Lo K.W., Yu J., Tse G.M.K. (2024). Cellular zinc metabolism and zinc signaling: From biological functions to diseases and therapeutic targets. Signal Transduct. Target. Ther..

[48] Fu T., Zhang S., Sheng Y., Feng Y., Wang C. (2020). Isolation and characterization of zinc-binding peptides from mung bean protein hydrolysates. Eur. Food Res. Technol..

[49] Maares M., Haase H. (2020). A guide to human zinc absorption: General overview and recent advances of in vitro intestinal models. Nutrients.

[50] Chen Y., Yang J., Wang Y., Yang M., Guo M. (2020). Zinc Deficiency Promotes Testicular Cell Apoptosis in Mice. Biol. Trace Elem. Res..

[51] Santos H.O., Teixeira F.J. (2020). Use of medicinal doses of zinc as a safe and efficient coadjutant in the treatment of male hypogonadism. Aging Male Off. J. Int. Soc. Study Aging Male.

[52] Pourhassanali N., Roshan-Milani S., Kheradmand F., Motazakker M., Bagheri M., Saboory E. (2016). Zinc attenuates ethanol-induced Sertoli cell toxicity and apoptosis through caspase-3 mediated pathways. Reprod. Toxicol..

[53] Li Y., Wang R., Li Y., Sun G., Mo H. (2022). Protective effects of tree peony seed protein hydrolysate on Cd-induced oxidative damage, inflammation and apoptosis in zebrafish embryos. Fish Shellfish Immunol..

[54] Asgharzadeh F., Roshan-Milani S., Fard A.A., Ahmadi K., Saboory E., Pourjabali M., Chodari L., Amini M. (2021). The protective effect of zinc on morphine-induced testicular toxicity via p53 and Akt pathways: An in vitro and in vivo approach. J. Trace Elem. Med. Biol. Organ Soc. Miner. Trace Elem. (GMS).

[55] Yirong C., Shengchen W., Jiaxin S., Shuting W., Ziwei Z. (2020). DEHP induces neutrophil extracellular traps formation and apoptosis in carp isolated from carp blood via promotion of ROS burst and autophagy. Environ. Pollut..

[56] Wang X., Simpson E.R., Brown K.A. (2015). p53: Protection against Tumor Growth beyond Effects on Cell Cycle and Apoptosis. Cancer Res..

[57] Yu G., Luo H., Zhang N., Wang Y., Li Y., Huang H., Liu Y., Hu Y., Liu H., Zhang J. (2019). Loss of p53 Sensitizes Cells to Palmitic Acid-Induced Apoptosis by Reactive Oxygen Species Accumulation. Int. J. Mol. Sci..

[58] Wu Y., Wang J., Zhao T., Chen J., Kang L., Wei Y., Han L., Shen L., Long C., Wu S. (2022). Di-(2-ethylhexyl) phthalate exposure leads to ferroptosis via the HIF-1α/HO-1 signaling pathway in mouse testes. J. Hazard. Mater..

[59] Matsumoto I., Mizuno Y., Seno N. (1979). Activation of Sepharose with epichlorohydrin and subsequent immobilization of ligand for affinity adsorbent. J. Biochem..

[60] Caetano-Silva M.E., Simabuco F.M., Bezerra R.M.N., da Silva D.C., Barbosa E.A., Moreira D.C., Brand G.D., Leite J.R.S.A., Pacheco M.T.B. (2020). Isolation and Sequencing of Cu-, Fe-, and Zn-Binding Whey Peptides for Potential Neuroprotective Applications as Multitargeted Compounds. J. Agric. Food Chem..

[61] Wang Q.L., Xiong Y.L. (2018). Zinc-binding behavior of hemp protein hydrolysates: Soluble versus insoluble zinc-peptide complexes. J. Funct. Foods.

[62] Mather J.P. (1980). Establishment and characterization of two distinct mouse testicular epithelial cell lines. Biol. Reprod..

[63] Du R., Li W., Li J.W., Zeng S., Chen Z.Q., Gao J.L., Zheng H.N., Lin H.S., Cao W.-H. (2024). Dynamic changes of zinc chemical speciation and zinc-containing peptides release in oysters (*Crassostrea hongkongensis*) during enzymatic hydrolysis. Food Biosci..

[64] Leaver-Fay A., Tyka M., Lewis S.M., Lange O.F., Thompson J., Jacak R., Kaufman K.W., Renfrew P.D., Smith C.A., Sheffler W. (2011). ROSETTA3: An object-oriented software suite for the simulation and design of macromolecules. Methods Enzym..

[65] Kang N., Heo S.Y., Kim E.A., Cha S.H., Ryu B., Heo S.J. (2023). Antiviral effect of fucoxanthin obtained from *Sargassum siliquastrum* (Fucales, Phaeophyceae) against severe acute respiratory syndrome coronavirus 2. Algae.

[66] Kang N., Kim E.A., Heo S.Y., Heo S.J. (2023). Structure-based in silico screening of marine phlorotannins for potential walrus calicivirus inhibitor. Int. J. Mol. Sci..

[67] Li C., Bu G., Chen F. (2020). Preparation and structural characterization of peanut peptide-zinc chelate. CyTA-J. Food.

[68] Sun X., Saleh A.S.M., Wang Z., Yu Y., Li W., Zhang D. (2024). Insights into the interactions between etheric compounds and myofibrillar proteins using multi-spectroscopy, molecular docking, and molecular dynamics simulation. Food Res. Int..

[69] Shen L., Tang X., Wei Y., Long C., Tan B., Wu S., Sun M., Zhou Y., Cao X., Wei G. (2018). Vitamin E and vitamin C attenuate di-(2-ethylhexyl) phthalate-induced bloodtestis barrier disruption by p38 MAPK in immature SD rats. Reprod. Toxicol..

